# Evaluating the Usability of Inertial Measurement Units for Measuring and Monitoring Activity Post-Stroke: A Scoping Review

**DOI:** 10.3390/s25123694

**Published:** 2025-06-12

**Authors:** Aishwarya Shenoy, Manvir Singh Samra, Karen Van Ooteghem, Kit B. Beyer, Sherri Thomson, William E. McIlroy, Janice J. Eng, Courtney L. Pollock

**Affiliations:** 1 Rehabilitation Research Program of the Centre for Aging SMART, GF Strong Rehabilitation Centre, Vancouver, BC V5Z 2G9, Canada; aishshen@student.ubc.ca (A.S.); janice.eng@ubc.ca (J.J.E.); 2 Graduate Program in Rehabilitation Science, University of British Columbia, Vancouver, BC V6T 1Z3, Canada; 3 Department of Physical Therapy, University of British Columbia, Vancouver, BC V6T 1Z3, Canada; manvirsamrapt@gmail.com (M.S.S.); kvanooteghem@uwaterloo.ca (K.V.O.); 4 Department of Health Sciences and Kinesiology, University of Waterloo, Waterloo, ON N2L 3G1, Canada; kit.beyer@uwaterloo.ca (K.B.B.); slfry@uwaterloo.ca (S.T.); wmcilroy@uwaterloo.ca (W.E.M.)

**Keywords:** stroke, usability, wearable sensors, activity monitoring

## Abstract

Stroke is the most common cause of disability in adults, resulting in declines in overall activity. Inertial measurement units (IMUs) allow for the monitoring of activity patterns in various settings, informing clinical interventions and patient self-management. This review aimed to synthesize existing research evaluating the usability of IMUs for monitoring activity in people with stroke. This scoping review was conducted according to PRISMA guidelines. The MEDLINE, Embase, and CINAHL databases were searched for publications evaluating the usability of IMUs for monitoring activity post-stroke. Fourteen publications met the inclusion criteria. Most studies were conducted in chronic stroke with data collected in real-world conditions. Usability findings indicated that most stroke participants and clinicians reported a positive user experience; however many reported difficulties with devices due to stroke-related impairments. Importantly, the interpretation of this finding was impacted by poorly reported impairments of stroke participants. Only a few studies evaluated end-user experiences (people with stroke and clinicians) in interpreting and engaging with feedback based on data from IMUs. Future usability studies in stroke populations should aim to understand participant characteristics that influence device engagement, with a focus on user experience in interpreting device-generated metrics.

## 1. Introduction

After a stroke, it is crucial for survivors to continue to engage in activity and movement to promote recovery and functional gains and prevent stroke recurrence. The rate of recurrence within the first year is reported to be 7.6% [1], with physical activity reported to be one of the strongest predictors of recurrent vascular events [2]. Current recommendations from the American Heart Association [3] suggest that individuals in the sub-acute phase of stroke recovery engage in 10 min of moderate-intensity activity 4 times per week and 20 min of vigorous activity 2 times per week, while individuals with chronic stroke (>6 months post-stroke) should participate in 150 min of moderate activity and 75 min of vigorous activity per week [4]. Unfortunately, stroke survivors are spending 75% of their day engaging in sedentary behavior [5]. This emphasizes the need to improve approaches to help stroke survivors monitor and manage their physical activity. Measuring physical activity volume and intensity post-stroke is commonly achieved using accelerometry and observation-based methods (e.g., behavioral mapping) [6]. However, the international consensus is to use accelerometry to measure physical activity post-stroke, as observational methods are time-consuming, labor-intensive, and cannot provide information on activity intensity or patterns in daily life, outside of clinical and hospital settings [6].

Accelerometers and inertial measurement units (IMUs), which include gyroscopes and magnetometers in addition to accelerometers, are small, non-invasive electronic devices that can be worn on different anatomical locations on the body. Extensive research has been performed to evaluate how data from accelerometers and IMUs can be used to provide measures for assessing gait [7,8], physical activity [9,10], sleep [5,10], and upper limb use [11,12], making them ideal for capturing longitudinal activity data. The metrics generated from these devices can inform clinical assessment and provide patients with the opportunity for self-evaluation and self-management in the real world [13,14,15,16].

Given the potential for positive impacts in stroke rehabilitation, it is essential to ensure that wearable devices are user friendly to promote both clinician and patient adoption and reduce device abandonment [17]. Collecting end-user (i.e., patients, clinicians, and caregivers) experiences and insights regarding device usability and application to everyday life is crucial for understanding clinician and patient needs. Analyzing device usability can be performed by evaluating users’ perspectives on a range of features including comfort, safety, durability, reliability, esthetics, and engagement [18,19]. The International Organization of Standardization (ISO) 9241-11 framework [20] is the most commonly cited usability framework of usability for wearable devices [21,22]. ISO defines usability as “the extent to which a product can be used by specified users to achieve specified goals with effectiveness, efficiency and satisfaction in a specified context of use” [20]. Effectiveness refers to the accuracy and completeness with which users achieve specified goals; efficiency relates to the amount of resources required by participants to achieve the pre-specified goals [23]; and satisfaction pertains to the user’s experience interacting with the system, inclusive of the data provided to the user. The framework highlights the importance of considering the **context of use**, which includes user characteristics, tasks, equipment, and the physical and social environment [20].

Context of use is especially important in stroke recovery due to the heterogeneity of the population, with significant variability observed in the level of impairment and rate of recovery. Post-stroke hemiparesis is experienced by 80% of individuals acutely and 40% chronically [24,25], significantly impacting the ability for stroke survivors to perform activities of daily living (ADLs) and maintain functional independence [26]. Stroke-related impairments, including spasticity and pain, can also impact ADL performance, upper limb reaching, grasping, and pinching, the latter of which may result in improper or inconsistent device wear and data inaccuracies [27,28]. Both cognitive [29] and communication (e.g., aphasia) [26] impairments can also impact the ability to interact and engage with wearable devices and the feedback they provide.

Previous reviews have explored the clinometric properties of wearable sensors [7,30]. To date, reviews exploring the application of IMUs in stroke rehabilitation have focused on their reliability for gait assessment [30], their usefulness in assessing motor function of the upper extremities [31], activity recognition [32], the quantification of activity (upper limb and general activity) [33], and sensor placement [8]. To our knowledge, this is the first scoping review to summarize the literature which has examined the usability of IMUs in the stroke population to monitor physical activity, upper extremity use, gait, or sleep. The aim of this scoping review is to synthesize existing research that evaluates the usability of inertial measurement units (IMUs) for monitoring activity in people with stroke. Specifically, it aims to summarize user experience in the context of the following: (1) participant and clinical characteristics; (2) methods of data collection, including the following: setting, device systems, and measures used for activity monitoring; and (3) methods of usability assessment and key usability findings.

## 2. Materials and Methods

### 2.1. Study Design

A scoping review was conducted, as it was the most appropriate methodology for summarizing evidence in the emerging area of IMU usability for monitoring activity in people with stroke and for identifying gaps in the literature related to the usability assessment of these devices. Given the exploratory nature of this review, the protocol was not registered; however, an established scoping review framework [34] was adhered to, ensuring methodological rigor. Full transparency was maintained through detailed reporting of the methodology within this manuscript. This study followed the Preferred Reporting Items for Systematic Reviews and Meta-Analyses Extension for Scoping Reviews (PRISMA-ScR) reporting guidelines (Appendix A).

### 2.2. Search Strategy

A search strategy was developed alongside an experienced health sciences librarian (CAB) and a series of preliminary searches were included to identify studies and key terms relevant to the study questions. The search strategy was applied to the MEDLINE, Embase, and CINAHL databases with an open-ended search date of March 2025 (Appendix B for Search Strategy). The reference lists of papers that made it to full-text screening were hand-searched for relevant articles.

### 2.3. Study Selection

Studies identified by the initial search were imported into a review management software (Covidence: “https://www.covidence.org accessed on 20 March 2025”). Following deduplication, two authors (AS and MSS) reviewed all titles and abstracts to select studies for full-text review using the eligibility criteria (Table 1). Articles which progressed to full-text review were independently assessed by AS and MSS and when a disagreement was present, a third author (CLP) was included for consensus.

### 2.4. Data Extraction

A data extraction tool was developed using previous reporting frameworks and published reviews as guidance [18]. Participant characteristics were extracted to inform the clinical context in which the usability of IMUs has been studied. The following characteristics were included to describe the context of use: type of stroke, stroke chronicity, level of mobility required to participate, level of physical and cognitive impairment, participant age, biological sex or gender, and number of participants. Details regarding data collection methods were extracted to understand the research design and IMU implementation, which included the following: year of publication, collection setting (e.g., free-living, lab, hospital), movement of interest (e.g., gait, physical activity, upper limb), device characteristics (e.g., system and type of sensor), number of sensors, wear location, and wear adherence. The following characteristics were extracted to assess the usability methods and findings: participants involved in usability assessment, usability aims, usability assessment methods, and key usability findings. The latter was coded to align with the three factors from the ISO 9241-11 definition of usability (effectiveness, efficiency, and satisfaction) [20] and were recorded in the usability assessment methods column. Data was extracted independently by two reviewers (MSS and AS). Any discrepancies were resolved by discussion with CLP for consensus.

### 2.5. Data Synthesis

Descriptive statistics (e.g., mean, standard deviation, frequency counts) were used to summarize participant and study characteristics. The lack of standardized assessments for usability made it difficult to provide descriptive statistics for close-ended, study-generated usability questionnaires. Usability findings from studies utilizing qualitative approaches (i.e., mixed methods, qualitative) were analyzed systematically by consolidating the results to identify recurring themes and similarities across studies.

## 3. Results

### 3.1. Search Results

**Figure 1:**
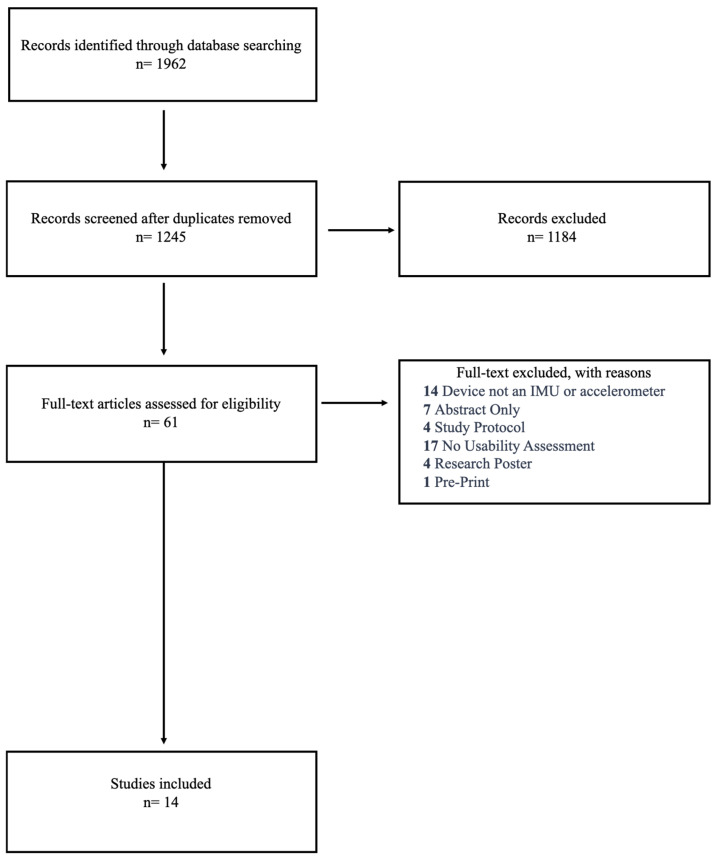
Flow diagram of study selection process.

The search identified 1962 articles. After deduplication, 1245 references were available for title and abstract screening, of which 1184 were excluded due to not being within the context of the research question. A total of 61 references moved onto full-text screening, of which 47 references did not meet the inclusion criteria for various reasons, leaving 14 articles for the scoping review (Figure 1).

### 3.2. Aim 1: Participant and Clinical Characteristics of Usability Studies

Participant and clinical characteristics are displayed in Table 2. A total of 314 participants were included across all studies (*n* = 14), ranging from 4 to 44 stroke participants and 13 to 15 health practitioner participants for each study. The average reporting age of the stroke participants was 60.7 ± 6.4 years, with one study that did not report participant age [35]. Most studies reported participant sex characteristics, with three studies reporting participant gender [36,37,38]. Across all studies there were 111 males, 83 females, 56 men, 32 women, 2 non-binary individuals, and 1 transgender man, with two studies not reporting the sex or gender of their participants [35,39]. Of the remaining studies, most participants had ischemic stroke (*n* = 143), followed by hemorrhagic (*n* = 37) and transient ischemic attack (TIA) (*n* = 10). Most studies included people with chronic stroke (>6 months post-stroke) (*n* = 10) [36,37,38,39,40,41,42,43,44,45], while two studies [46,47] were in sub-acute populations (>1 week and <6 months post-stroke), and two studies [35,48] did not provide information on the stage of stroke recovery. Two studies [42,45] excluded people with aphasia and two studies [37,44] modified their materials to include people with aphasia; the remaining studies did not report whether individuals with aphasia were included. Only one study provided information on lesion location [41].

Half of the studies (*n* = 7) required participants to independently ambulate with or without a gait aid [37,38,40,41,44,46,47], four studies included participants who were non-ambulators [46] or required physical assistance/supervision [35,36,42], and two studies [43,48] did not report the level of independent mobility required to participate. Studies (*n* = 3) that provided mobility characteristics [36,37,41] reported participant mobility ranging from limited to independent community ambulation. The Fugl-Meyer Assessment (FMA)—Upper Extremity was the most common method for assessing post-stroke impairment (*n* = 7) [36,37,38,39,42,43,46], while six studies did not assess the level of impairment for their study population [35,40,44,45,47,48]. For those who reported the impairment level, upper extremity impairment ranged from mild to moderate. Five studies provided measures of cognitive function [36,37,38,42,44], five studies mentioned that individuals with cognitive impairment were excluded [35,41,43,45,48], and the remaining studies did not mention the level of cognitive functioning required to participate [39,40,46,47].

### 3.3. Study Contexts, Activities Monitored, and Wearable Sensor Configurations

The contexts for data collection, activities monitored, and device configurations are presented in Table 3. The year of publication ranged from 2008 to 2024, with the majority of publications originating after 2018 (*n* = 12). It was most common for studies to use accelerometers only (*n* = 7) [35,38,40,41,43,45,46,47]. Of the eight studies that used IMUs, five used both gyroscope and accelerometer, and the inclusion of the gyroscope was typically for gait monitoring (*n* = 4). The number of sensors on the body ranged from one to five, with seven different anatomical locations, including the wrist, index finger, ankle/foot, leg/thigh, hip, trunk, and chest. Some studies used multiple anatomical locations (*n* = 5) [35,36,37,46,48], with the ankle and wrist being the most common combination for studies using multiple locations (*n* = 4) [36,37,46,48]. Devices were used to measure upper limb movement and performance (*n* = 7) [36,37,38,39,42,43,46], lower limb performance and function inclusive of gait (*n* = 8) [35,36,37,40,41,42,44,46], physical activity (*n* = 3) [45,47,48], and postural transitions (*n* = 1) [40]. Five of these studies [36,37,40,42,46] conducted measurements in more than one activity domain (i.e., gait and upper limb). The most common measurement for upper limb activity was active movement time (*n* = 5) [36,37,38,43,46]. Step count was most commonly used for lower limb performance (*n* = 4) [35,36,37,40] and for the three studies that examined physical activity, only one specified the measure used [45], which was mean vector magnitude.

For all methodologies, free-living was the most common setting of collection (*n* = 9) [37,38,40,42,43,44,45,46,47], with the remaining occurring during in-patient stay (*n* = 2) [35,48] and in a laboratory setting (*n* = 3) [36,39,41]. The device wear time in free-living settings ranged from 30 min to 2 weeks, with most studies monitoring activity over multiple days (*n* = 7). In studies that specified the required wear time, participants generally followed the instructions. However, five studies did not report the expected daily wear time [35,40,45,46,47], making it difficult to assess wear compliance.

### 3.4. Methods of Usability Assessment and Usability Findings

Assessment methods and key usability findings for the included studies are presented in Table 4. Studies were categorized according to the evaluation methodology (quantitative, qualitative, and mixed methods) to account for how the chosen methodology influenced the reported usability findings. All fourteen included studies assessed usability in people with stroke, but three also included clinicians [39,43,48]; two of these included occupational therapists [39,43] and one included multidisciplinary clinical participants (physicians, nurses, and therapists) [48]. In terms of the study design, more than half were quantitative (*n* = 8) [35,36,39,40,41,42,46,47], four studies used mixed methods [37,38,45,48], and the remaining were qualitative (*n* = 2) [43,44].

For quantitative studies, the most common study aim was to assess the acceptability of [36,40,42,46,47] and user satisfaction [41,42] with the devices, with one study evaluating both aspects simultaneously [42]. With respect to the ISO framework, five studies assessed effectiveness [35,36,39,40,41], six assessed efficiency [35,36,39,40,41,46], and seven assessed satisfaction [35,36,40,41,42,46,47], with four [35,36,40,41] assessing all three aspects of usability as defined by the ISO framework. All studies evaluated usability with questionnaires, with most creating their own (non-standardized) questionnaire (*n* = 6) [35,36,39,40,46,47]. Study-generated questionnaires varied in response types, including dichotomous answers (i.e., yes/no) and Likert scales for rating comfort and experience, further complicating result comparisons. Two studies employed established usability and user experience questionnaires, such as the Technology Acceptance Model (TAM) [42] and the Quebec User Evaluation of Satisfaction with Assistive Technology (QUEST) [41]. Most stroke participants reported positive experiences with the sensors used with respect to comfort. Although it was a common finding for patients to report that donning and doffing sensors was not an issue, participants in two studies reported difficulties with these tasks [36,46]; however, the reasons for these difficulties were not specified. It should be noted that despite Schließmann et al. [41] using quantitative methodology, they reported usability findings which would not have been captured through their questionnaire (participant feedback reported without specified qualitative methodology). For example, they reported that a patient with aphasia would have preferred if the verbal feedback from the system was provided as simple sounds rather than polysyllabic words.

In qualitative studies, the study aims typically involved examining receptiveness and attitudes towards the devices, which included looking at the utility of the data that comes from wearable sensors [43]. With respect to the ISO framework, all studies evaluated all three aspects of usability through the use of interviews. While the usability findings largely aligned with those of quantitative studies, the qualitative studies provided a deeper understanding of the underlying reasons behind patient and clinician comfort and satisfaction with the devices. For example, people with stroke reported that difficulty with attaching and removing devices was due to their size or the need for help putting them on [44]. This concern was echoed by occupational therapists, who highlighted that stroke-induced physical impairments might hinder the ability to don and doff sensors [43]. With respect to interacting with the devices, Nieboer et al. [44] reported that activity feedback played an important role in realizing the importance of physical activity. These thoughts were shared by people with stroke in Jung et al. [43], who expressed a preference for choosing the platform (e.g., phone or desktop) on which they reviewed their data, in order to better integrate it into their daily lives. Health practitioners from Jung et al. [43] believed that the data from the sensors could be used to personalize therapy sessions; however, they indicated a need for normative data (stroke or age matched controls) to guide interpretation prior to using it with patients.

For mixed methods studies, two studies assessed effectiveness and efficiency [38,45] and three assessed satisfaction [37,38,48], with one [38] assessing all three aspects of usability as defined by the ISO framework. Usability findings were consistent with what was reported in the quantitative and qualitative studies. People with stroke preferred real-time feedback and daily summaries that highlighted their progress toward individual goals, which could serve as motivation to support behavioral change [37]. They also reported that the feedback was easier to understand once they were oriented to it, with mobility metrics easier to understand compared to upper limb metrics [37], but the reasons for the difficulty in understanding upper limb metrics were not explored. These studies also suggested that using data in conjunction with a therapist would be more beneficial, as therapists could promote accountability and help patients maintain their activity levels through support and guidance [37]. One study that included healthcare practitioners reported mixed views on the use of wearable sensors and artificial intelligence in healthcare delivery [48]. Therapists saw it as a valuable opportunity, while doctors and nurses were less enthusiastic. In line with usability findings from the quantitative and qualitative studies, two studies [38,48] noted that the severity of motor impairment could impact adherence to wearing the sensors, as individuals with severe impairments might struggle to don and doff the sensors independently.

For the studies that included clinicians, many were willing to use a wearable sensor system in the clinic [39,43,48] and believed it would be beneficial for encouraging patients to perform home-based exercises [39]; however, no studies looked at clinician satisfaction with the metrics that were produced by the devices for assessing patient activity and recovery. Across all quantitative studies, only two [39,41] examined the usability of the mode of feedback. Lee et al. [39] reported that most participants with stroke preferred a combination of sound and vibration. Schließmann et al. [41] reported that stroke participant satisfaction with the feedback design ranged from ‘not satisfied’ to ‘very satisfied’, but provided no details about which aspects of the design left some participants unsatisfied.

## 4. Discussion

This scoping review provides an overview of studies looking at the usability of IMUs for monitoring and measuring activity in individuals with stroke. All studies included in this review gave participants the opportunity to interact and engage with the devices prior to evaluating usability. Findings suggest that stroke-related impairments (e.g., aphasia, motor impairment) may play a role in a patient’s ability to use the device and the feedback it provides [35,36,38,41,43,44,46,48], highlighting the importance of reporting these characteristics to provide information on context of use. More than half of the studies (8/14) included in this review measured activity in “free-living” (real-world) settings, most commonly monitoring upper limb activity and gait in free-living environments, reflecting a growing emphasis on capturing real-world activity in addition to clinical performance. Overall, participants, both clinicians and people with stroke, reported positive experiences with IMUs to monitor and measure activity. Studies incorporating qualitative methodologies (e.g., interviews, focus groups) and involving all forms of end-users (i.e., clinicians, patients, and caregivers) can provide richer insights into important aspects of use such as how impairments impact effectiveness and efficiency of and satisfaction with devices used [38,43,44,48]. This review identified a gap in assessing the usability with the activity feedback provided by these devices, which have the potential to support clinicians in decision-making and help people with stroke to self-manage their day-to-day activity levels.

Inconsistent reporting of stroke-specific participant characteristics impacts the interpretation of findings in the context of reported usability across the spectrum of stroke recovery (mild to severe stroke; acute to chronic stage of recovery). At least half of the studies did not report the level of sensorimotor and cognitive impairment, or the presence of aphasia, despite usability findings highlighting these as important factors for understanding an individual’s ability to effectively and efficiently engage with the devices [36,38,41,43,44,48]—two core dimensions of usability as defined by the ISO framework. Inability to don and doff devices may result in improper wear, compromising data quality and resulting in inaccurate feedback, ultimately reducing the effectiveness of the device in supporting rehabilitation or activity monitoring goals. Additionally, most of these studies were conducted in people with chronic stroke, who are likely to have plateaued in recovery post-stroke [49]. The usability of IMUs is not highly explored in the sub-acute phase of recovery, a period characterized by a substantial improvement in functional gains [49,50]. The rate of change in recovery and function may have significant impacts on an individual’s ability to effectively and efficiently use a device. Using IMUs in the sub-acute population was recently recommended by the Stroke Recovery and Research Roundtable [51], as these devices can objectively capture functional changes in clinic and real-world settings during this critical recovery period. The transition from inpatient rehabilitation to discharge home takes place during the sub-acute phase and is reported to be a challenging period due to the reduced structure and increased self-management. During this transition, usability becomes especially relevant, as the technology must be intuitive, acceptable, and non-burdensome to support ongoing engagement [52].

The setting of implementation (context of use) can impact the usability of a device for both clinicians and patients. This context shapes who interacts with the device, how it is used, and what features are prioritized. For example, in clinical settings, devices are typically operated by trained professionals, so ease of use for patients is less critical, and the metrics are designed to inform clinical decision-making. In contrast, in free-living environments, patients must independently wear, interpret, and respond to feedback from the device—making intuitive design, comfort, and minimal user burden essential for sustained engagement. Recognizing how usability needs differ by setting is particularly relevant given recent trends in the literature. Studies published in the last 5 years were mostly conducted in free-living settings, suggesting a shift toward measuring activity that better reflects the day-to-day function of individuals. Notably, within studies collected in free-living settings, a shift towards monitoring multiple domains of activity is suggested, including gait, upper limb use, and balance [36,37,40,42,46]. This reflects the ability to more comprehensively measure the impact of stroke and likely reflects the continuing advancement in sensor development (e.g., data logging, computational power) and data analytics incorporating multiple sensors [10]. Despite real-world activity monitoring becoming more prevalent, compliance with wearing devices throughout the day for multiple days is poorly reported, with only a few studies mentioning participant experiences in wearing the devices for extended periods of time. Previous research has reported lower daytime wear of IMUs among individuals with neurodegenerative disorders [53] and reduced nighttime wear in those with chronic obstructive pulmonary disorder (COPD) [54]. These findings suggest that compliance varies across chronic conditions, likely due to differences in symptom presentation and factors affecting engagement with devices. This underscores the importance of future stroke studies providing detailed reporting of participant characteristics which may impact an end-users’ experiences in using devices over extended periods of time.

Metrics provided by the studies in this review to measure upper and lower extremity activity varied significantly, reflecting differences in the assessment and treatment of post-stroke impairments between the upper and lower extremities. For the upper extremity, clinicians have expressed a preference for measures that reflect both the quantity and quality of upper extremity movement—such as smoothness and compensation [15,55,56]. Derived metrics are sensor dependent. Devices with only accelerometers are suggested to primarily capture movement quantity, such as active movement time/duration of use [57], whereas the use of gyroscopes in addition to accelerometers allows for detailed assessments of movement quality, including metrics like smoothness [58] and the presence of compensation [12,58].

A majority of the study methods evaluating usability included single study questionnaires and standardized assessments which rely on dichotomous (i.e., Yes/No) and Likert scale responses. While valuable to quantify usability, these scales do not capture nuanced end-user experiences. For example, the study-generated questionnaire used by Lau et al. [47] revealed that 23% of participants removed sensors during data collection, but the response types did not provide insights into the reason for removal. Alternatively, studies that took a mixed methods approach where they used a standardized assessment in conjunction with a qualitative component (e.g., interviews) were well positioned to compare their quantitative results with other studies while also being able to obtain a nuanced understanding of participant experiences related to wearing, using, and taking off the device—all important factors for assessing the user experience of someone living with stroke [21,55,59]. Utilizing qualitative methodologies allows for the in-depth exploration of user needs, motivations, and preferences compared to quantitative methodology [18,60]. Meanwhile, mixed methods approaches are reported to provide a holistic picture of system usability for assistive technologies [61,62,63]. Future studies aiming to evaluate the usability of devices in people with stroke should consider adopting a mixed methods approach to obtain a more robust and nuanced understanding of end-user experiences by leveraging the strengths of both quantitative and qualitative approaches [64]. Standardized assessments or questionnaires can be used to provide summative measures that can evaluate usability and enable comparisons across studies. These scores can then be used to help identify specific areas for further qualitative exploration. However, it should be noted that a comparison of the scores from standardized assessments and questionnaires will be limited to studies that share similar participant characteristics.

End-user experiences of the effectiveness and efficiency of or satisfaction with activity feedback from the devices to support self-management of activity levels and clinical decision-making remain underexplored, despite stroke survivors and clinicians expressing interest in personalized, real-time feedback for assessing their performance [35,37,39,41,43,44,46]. Of these, only five studies [37,39,41,43,44] evaluated participant experiences with device feedback but provided limited insight into its effectiveness for supporting self-management, and none investigated clinicians’ perspectives on the metrics’ ability to effectively and efficiently support clinical decision-making. This is an important area that needs further exploration, as some of these metrics might require clinicians and patients to be educated and oriented towards its use prior to implementation, especially for patients who may have some form of cognitive impairment as a result of their stroke. Engaging both clinicians and patients in device and metric development ensures that clinicians receive the necessary information to assess recovery and engagement [65], facilitating integration into clinical practice, while also providing patients with meaningful feedback to support the self-management of activity. As a result, it remains unclear whether usability is primarily influenced by the type of device, the nature of the feedback it provides, or a combination of both.

Some limitations of this review are worth noting. This review did not report on proprioceptive and spatial impairments, which are commonly experienced by individuals post-stroke and may influence engagement with wearable devices. The decision to focus solely on cognitive and physical impairments was made due to the limited availability of studies addressing how proprioceptive and spatial impairments affect the use of wearable technologies in stroke rehabilitation. Additionally, the definition and terminology with respect to usability is inconsistent across studies aiming to explore user experiences, which can impact search results [18]. The broad and varied use of search terms served to address this limitation. Lastly, other sensors (i.e., electromyography (EMG), pressure sensors, and heart rate monitors) are also capable of measuring activity and performance but were not included in this review as IMUs and accelerometers are currently the most utilized devices for measuring and monitoring activity in people with stroke [66].

## 5. Conclusions

This scoping review summarizes the literature which has examined the usability of IMUs in the stroke population for monitoring physical activity, upper extremity use, gait, or sleep. Most usability studies have been conducted in the chronic stroke population, where user needs and abilities can differ from those in the sub-acute phase of recovery, identifying an important future direction for research. Underreported participant characteristics—such as the level of sensorimotor impairment and other stroke-related impairments (e.g., aphasia)—limits the ability to interpret how these factors may influence the effectiveness, efficiency, and satisfaction associated with device use, resulting in an incomplete understanding of usability in the stroke population. There has been growing interest in evaluating the usability of devices for the measurement of free-living behavior post-stroke, suggesting a growing interest in measuring activity outside the clinic, where device-generated data could support the monitoring of recovery and support the self-management of activity levels. However, certain aspects of device usability remain underexplored. In particular, the effectiveness and efficiency with which clinicians can utilize device metrics in clinical settings, as well as the satisfaction and ability of patients to understand and integrate these metrics into their daily lives, require further investigation.

## Figures and Tables

**Table 1:** Inclusion and exclusion criteria used to assess eligibility of studies.

Inclusion Criteria
Adults (age 18+) with stroke (first ever or recurrent, at any stage of recovery—acute, sub-acute and chronic) and/or healthcare professionals working in stroke rehabilitation Using IMUs and/or accelerometers to monitor/measure any type of activity (i.e., upper limb activity, physical activity, sleep, gait) Study methods: quantitative, qualitative and mixed methods Assessment of usability—including terms such as ‘feasibility’ and ‘wearability’ to capture alternative terminology used in the literature
Exclusion Criteria
Clinometric properties (e.g., validation and/or reliability studies of sensor-based metrics) without measurement of user experience Reviews/study protocols/preprints Use of device data to assess treatment outcome(s)

**Table 2:** Participant characteristics showing clinical context in which usability of IMUs and accelerometers have been assessed in people with stroke.

Study	Type of Stroke	Stroke Chronicity	Mobility	Impairment Mean (SD)	Time Since Stroke (Months) Mean (SD)	Number of Stroke Participants (Sex or Gender)	Age of Participants Mean (SD)
[45]	Not Provided	Chronic	Walking independently w/or w/o gait aid	Physical: Not Provided Cognitive: Not Provided *	Not Provided	20 (10 male/10 female)	72 (7.1)
[40]	4 Hem; 6 Isch	Chronic	Walking independently w/or w/o gait aid	Physical: Not Provided Cognitive: Not Provided	42 (25)	10 (3 male/7 female)	70 (8)
[41]	2 Hem; 8 Isch; 1 unresolved	Chronic	Able to walk w/or w/o gait aid for 20 min and ≥100 m for 6MWT 6MWT (meters): 356.6 (91.9)	Physical: ISNCSCI- Muscle Test: 21.8 (4.4) Cognitive: Not Provided *	65.9 (74.1)	11 (7 male/4 female)	56.8 (7.7)
[39]	Not Provided	Chronic	Not provided	Physical: FM-UE (/66) 37 (8) Cognitive: Not Provided	55.2 (66)	17 (not provided)	54.4 (10.1)
[46]	5 Hem; 21 Isch	Sub-acute	Walking independently w/or w/o supervision FAC 0–3 (13) FAC 4–5 (13)	Physical: FM-UE (/66) Median: 35 Q1–Q3: 15–50 Cognitive: Not Provided	1.87 (0.8)	26 (16 male/10 female)	55.4 (11)
[35]	Not Provided	Not Provided	FAC ≥ 2	Physical: Not Provided Cognitive: Not Provided *	Not Provided	13 (not provided)	Not provided
[42]	22 Isch	Chronic	Walking independently indoors w/o aid or with aid and supervision	Physical: FMA (/226) 110.4 (29.41) Cognitive: MMSE(/30) 26 (1.8) *	11.5 (2.4)	22 (15 male/7 female)	55.3 (8.6)
[48]	5 Hem; 29 Isch and 10 TIA	Not provided	Not provided	Physical: Not Provided Cognitive: Not Provided *	Not Provided	44 (22 male/22 female)	64 IQR (24–92)
[47]	12 Hem; 28 Isch	Sub-acute	Walking independently w/or w/o gait aid	Physical: Not Provided Cognitive: Not Provided	69.4 (38.9)	40 (23 male/17 female)	52.8 (7.38)
[43]	Not Provided	Chronic	Not provided	Physical: FM-UE (/66) 51 (11.2) Cognitive: Not Provided *	89.7 (52.5)	4 (2 male/2 female)	69.5 (5.9)
[44]	Not Provided	Chronic	Community dwelling w/or w/o walking aid; gait speed < 1 m/s	Physical: Not Provided Cognitive: MoCA 24.5 (4.2)	Range: 17–172	17 (13 male/4 female)	61.9 (18.8)
[37]	7 Hem; 21 Isch; 2 not reported	Chronic	Walking independently on 10 m walk test: Self-paced: 0.75 m/s (0.4)	Physical: FM-UE 41.2 (18) Cognitive: MoCA(/30) 24.7 (3.4) *	91.2 (54)	30 (18 men/11 women/1 non-binary)	58.6 (13.1)
[36]	Not Provided	Chronic	FAC ≥ 3 Gait speed: 0.64 m/s (0.39)	Physical: FM-UE (/66) 37.3 (17.6) Cognitive: MoCA(/30) 24.9 (3.8) *	78.7 (48.8)	30 (19 men/10 women/1 non-binary)	57 (10)
[38]	Not Provided	Chronic	Walking independently	Physical: FM-UE Median: 46.0 Range = 18–66 Cognitive: MoCA(/30) Median: 26 *	Median: 87.8 Range: (54–129)	30 (18 men/11 women/1 transgender man)	Median: 61.5 Range: 48.5–64.9

Abbreviations: FM, Fugl-Meyer; Hem, Hemorrhagic; Isch, Ischemic; ISNCSCI, International Standards for Neurological Classification of Spinal Cord Injury; FACs, Functional Ambulatory Categories; m/s, meters/second; 6MWT, 6-Minute Walk Test; TIA, Transient Ischemic Attack; MMSE, Mini Mental State Examination; MoCA, Montreal Cognitive Assessment. Acute = 1–7 days post-stroke; sub-acute = 7 days–6 months post-stroke; chronic = >6 months post-stroke. * Exclusion criteria mentioned cognitive impairment.

**Table 3:** Summary of study methods defining context of use, including year of publication, data collection setting, activity of interest, type of system used, number of sensors worn and their body locations, and measurement of wear adherence.

Study	Year	Setting	Activity of Interest	Device	Number of Sensors and Wear Location	Wear Adherence	
						Directed by Study Methodology	Reported Wear Time Mean (SD)
[45]	2008	Free-living	Physical Activity: Mean vector magnitude	System: RT3; 3-axis Sensors used: accelerometer	One sensor attached to a waist belt in a central back position	7 days (hours/day not specified)	11 h/day
[40]	2016	Free-living	Postural Transitions: Duration in seconds, and # of unsuccessful attempts Gait: Steps, speed, and duration	System: PAMSys (Biosensics LLC, MA, USA); 3-axis Sensors used: accelerometer	One on mid-sternal pocket located in a comfortable t-shirt	2 days (hours/day not specified)	2 days
[41]	2018	Lab	Gait: Stride length, stance or swing duration, or foot-to-ground angle	System: RehaGait; 9-axis Sensors used: accelerometer and gyroscope	Two sensors: One on each shoe, mounted to users’ shoes (lateral, just below ankle joint)	Three training sessions: Minimum of 22.5 min for each session	37.5 min (7.4 min)
[39]	2018	Lab	Upper Limb: Goal-directed and non-goal-directed movements	System: Shimmer; 6-axis Sensors used: accelerometer only for ADLs, gyroscope and accelerometer for rehabilitation exercises	Two sensors: One on each wrist	Worn during completion of motor tasks, ADL tasks, and rehabilitation exercises.	Not applicable
[46]	2019	Free-living	Upper and Lower Limb: Signal Magnitude Area (SMA) ratio for arms and legs, duration of arm and leg use	System: Shimmer^®^3; 3-axis Sensors used: accelerometer	Five sensors: One on each wrist, one on each ankle, and one on trunk	Two separate 48 h sessions on weekdays and over a weekend	Missing data for all five sensors (<20 h): 11 participants on weekdays; 19 participants on weekends
[35]	2020	In-patient setting	Gait: Speed, step count, and stride length	System: Shimmer^®^3; 3-axis Sensors used: accelerometer	Three sensors: Two on ankle and one under the mattress of participant at level of thorax	5–7 days (hours/day not specified—only worn during daytime, taken off prior to bedtime and when showering)	Median: 6 days 11.1 ± 2.0 h/day
[42]	2020	Free-living	Lower Limb: Abduction and adduction of the hip, knee and hip ROM (flexion-extension) Upper Limb: Flexion–extension of wrist, elbow, and shoulder Balance: Lateral trunk flexion and torsion of trunk	System: WeReha device; 9-axis Sensors used: specific sensors not specified	One sensor which could be placed on either trunk, leg, foot, or wrist depending on the exercise they are performing	During exercises proposed by WeReha (15–30 min) Note: Exact wear time not specified	Not provided
[48]	2020	In-patient setting	General Activity	System: Apple Watch Series 3; 9-axis Sensors used: accelerometer and gyroscope	Four sensors: One on each wrist, one on each ankle	24 h/day, 1 day	Range: 1.5–24 h
[47]	2022	Free-living	Physical Activity	System: activPAL; 3-axis Sensors used: accelerometer	One on anterior unaffected thigh	7 days (hours/day not specified)	7 days
[43]	2022	Free-living	Upper Limb: Duration and ratio of bilateral limb use	System: name not provided; 3-axis Sensors used: accelerometer	Two sensors: One on each index finger	8 h/day, 2 days	Not provided
[44]	2023	Free-Living	Gait: Speed, step length, and step height	System: (54 × 33 × 14 mm, 22 g, EXEL s.r.l, Bologna, Italy), and an app (“Stappy”) was installed on an Android smartphone (Mortorola Moto G 3rd generation); 6-axis Sensors used: accelerometer and gyroscope	Two sensors: One sensor on each shoe connected via Bluetooth to smartphone	Use during walks for a two-week period	4.5 days (3.9 days)
[37]	2023	Free-living	Upper Limb: Active movement time Gait: Step count and stance time	System: MiGo activity watch (FlintRehab); 6-axis Sensors used: accelerometer and gyroscope (gait)	Four sensors: One on each wrist and one on each ankle	12 h/day, 7 days	Not provided
[36]	2024	Lab	Upper Limb: Active movement time and movement counts Gait: Step count and stance time	System: MiGo activity watch (FlintRehab); 6-axis Sensors used: accelerometer and gyroscope (gait)	Five sensors: One on each wrist, one on each ankle, and one on hip	Worn during completion of UL and mobility standardized assessments	Not applicable
[38]	2024	Free-living for monitoring arm use and lab for assessment	*Upper Limb:* Time of active movement for each arm, arm use ratio	*System:* MiGo activity watch (FlintRehab); 6-axis *Sensors used:* accelerometer	Two sensors: One on each wrist	12 h/day, 7 days	12.6 (0.2) h/day

**Table 4:** Summary of usability assessments and key findings. ISO usability indicates if measure captures the following: 1. effectiveness, 2. efficiency, or 3. satisfaction.

Study	Participants	Study Aim	Usability Assessment Methods	Usability Findings
Quantitative				
[40]	10 with stroke	Determine the acceptability of wearing the PAMSys equipment (i.e., t-shirt with Velcro pocket closure for sensor) for 48 consecutive hours	Method: Phone call interview with study-generated questionnaire * Response Type: Yes/No ISO Usability: 1,2,3	- 100% commented that the PAMSys was comfortable, lightweight, enjoyable, and easy to wear - Patients had no challenges with the equipment - None reported any difficulty with the PAMSys while sleeping; with removing or putting it back on for showering or changing clothes; or that it became wet or dirty
[41]	11 with stroke	Evaluate user satisfaction of the RehaGait real-time feedback system	Method: Modified Quebec User Evaluation of Satisfaction with Assistive Technology (QUEST) * ISO Usability: 1,2,3	- Users satisfied with the therapeutic efficacy of the system - One individual with a stroke associated with aphasia suggested the implementation of an optional feedback output using simple sounds instead of polysyllabic words - Stroke participants valued efficacy and feedback design more than safety, comfort, and reliability - Satisfaction with feedback design ranged from not satisfied to very satisfied - Satisfaction with efficacy ranged from quite satisfied to very satisfied
[39]	17 with stroke 13 occupational therapists	Evaluate the appropriateness of the envisioned technological approach for stroke survivors and occupational therapists (OTs)	Method: Study-generated questionnaire Response Type: Yes/Neutral/No ISO Usability: 1,2	Stroke - 76.5% of stroke survivors were willing to use the system and the reminders encouraging the use of the stroke-affected upper limb - 82.3% of stroke survivors expressed confidence that the system would help increase the use of their stroke-affected upper limb - For mode of feedback, 29.4% preferred visual message, 11.8% sound, 23.5% vibration, and 35.3% combination of sound and vibration OTs - 91.7% willing to use the system in their clinical practice - 100% thought that the device would help encourage patients to perform home-based exercises - 61.5% wanted periodic email alerts about their patient’s performance - 100% expressed interest in a system that can provide patients with reminders throughout the day to encourage the use of the stroke-affected upper limb
[46]	26 with stroke	Feasibility of sensor measurement in terms of comfort, acceptance, and management in the clinical setting	Method: Study-generated questionnaire (close-ended) Response Type: 5-point Likert scale ISO Usability: 2,3	- 50% forgot to wear sensors - 33% reported the trunk sensor to be uncomfortable - 31% reported difficulty with the Velcro straps - Few participants were interested in the results from their wear - 1 participant’s data was excluded due to non-adherence related to cognitive impairment - Sensors were well tolerated overnight, with 5 participants reporting that the LED lights from the sensors were disturbing
[35]	13 with stroke	Assess user experience of a sensor-based platform which can continuously measure gait and sleep	Method: Study-generated questionnaire * Response Type: Yes/No and 0–10 scale ISO Usability: 1,2,3	- 9/12 participants required assistance to don/doff sensors - Easiness of use/10 (mean: 8, range 6–10) - No participants were bothered or impeded in their activities by the IMUs Findings below are for the whole group; the study did not specify stroke-specific findings: - 7/24 experienced increased cognitive load (needing to remember to put on sensors and placement of sensors) - 20/24 willing to wear sensors again - 18/24 interested in data for activity performance (*n* = 8), progression (*n* = 7), and gait parameters (*n* = 6) - Participants suggested straps be refined, future devices should be waterproof, and reduced flashing LEDs on the IMUs
[42]	22 with stroke	Satisfaction and acceptance of WeReha device by patients	Method: Technology Acceptance Model (TAM) * ISO Usability: 3	- TAM A (/49) = perceived ease of use 42.455 (6.131) - TAM-B (/42) = perceived utility 35.727 (6.475) - TAM-C (/35) = attitude toward new technologies 22.818 (3.972) - TAM-D (/28) = mean attitude toward the use of new technologies 23.955 (5.376) - Participants with higher TAM scores derived the greatest benefit from the device
[47]	40 with stroke	Acceptability of multimodal ambulatory monitoring system to assess daily activity and health-related symptoms among community-dwelling survivors of stroke	Method: Study-generated Likert scale questionnaire * Response Type: 3- and 5-point scale; Yes/Maybe/No ISO Usability: 3	- 97.5% rated overall experience as positive - 77% of participants never removed the accelerometer during the study protocol - Most participants were highly satisfied with and confident in using the study technology (mean satisfaction, 4.8/5; mean confidence, 4.7/5) and preferred it over traditional methods (i.e., retrospective self-reports) (mean preference, 4.7/5) - Participants reported low interference of accelerometer with their daily routine
[36]	30 with stroke	Assess the acceptability of MiGo in terms of ease of use, ease of donning and doffing, and esthetics	Method: Study-generated questionnaire * Response Type: 5-point Likert scale ISO Usability: 1,2,3	- All participants rated high comfort for each sensor - Most participants were easily able to don and doff the sensor on non-paretic side, with 4 unable to don the ankle sensors - 86.7% expressed willingness to wear all sensors daily - Time to don was the highest for the nonparetic wrist with inter-individual variability - Time to don either ankle sensor (paretic: 61.7 (52.8) seconds and nonparetic: 59.6 (62.3) seconds) was longer than the hip clip (13.6 (8.7) seconds)
Qualitative				
[43]	4 with stroke 15 occupational therapists	Examine stroke survivors’ and therapists’ receptiveness towards arm performance data and how they use the data	Method: Interview * ISO Usability: 1,2,3	Themes: Receptiveness of the ring sensors and arm movement data - 3/4 patients willing to wear ring sensors in daily living for a prolonged time if endorsed by clinicians - 1/4 felt like wearing sensor would not address his issues (pain) - Stroke survivors did not feel ashamed of wearing the ring in public - OTs reported that collection and reviewing of data from sensors would not be burdensome Opportunities for using patient-generated sensor data to personalize therapy - OTs reported that data from sensors would help with personalizing therapy - One OT cautious about using data to define goals as sensor gives amount of use and not quality movement - 3 OTs reported need for normative data (stroke or age-matched healthy individual) prior to use for goal setting with patients Potential ways to improve remote monitoring system to further support its translation - Size of sensor made it difficult to don and doff clothes/gloves and put their hands into pockets - OT concern about stroke-induced physical impairments and edema may hinder patients from wearing sensor - Stroke participants showed positive receptiveness to wearing sensor but preferred different ways for delivery of feedback (i.e., phone app, desktop)
[44]	17 with stroke	To assess attitudes towards “Stappy” in people after stroke practicing walking performance independently at home	Method: Interview * ISO Usability: 1,2,3	Themes: Active reminders Quality of feedback was positively appreciated as it helped participants be consciously aware of the importance of being physically active with the goal of improving their mobility. 2. Integrating Stappy into daily walking Apart from walking, participants also wanted to be able to use Stappy during daily activities. Some participants reported persistent feedback to be annoying when used during daily walking activities. 3. Ease of use Putting the sensors on the shoes was difficult for many due to the impairments caused by their stroke. App which provided feedback was easy to use. 4. Support by social environment The presence of social support when using “Stappy” seemed to play a role for the actual use of the system, especially for those with significant impairments. These participants required help to put the sensors on.
Mixed Methods				
[45]	20 with stroke	Investigate the utility of RT3 to measure physical activity in the free-living environment in adults with and without neurologic dysfunction	Method: Quantitative: Study-generated questionnaire * Response Type: 9-point scale Yes/Maybe/No Qualitative: Open-ended questionnaire * ISO Usability: 1,2	Findings below are for the whole group; the study did not specify stroke-specific findings: Utility Questionnaire - Most reported wearing the accelerometer every day to be acceptable, that they were not annoying to wear, they were able remember to wear them, and they did not interfere with daily life - 38% would wear the accelerometer again - 89% reported accelerometer as user-friendly Open-Ended Questions: - Positioning of the sensor on lower back was uncomfortable (especially when sitting or driving) - 6% found sensor to be too big - 15% said it was easy to wear - 36% worried that sensor would fall off - 25% reported keeping a diary was burdensome
[48]	44 with stroke 15 healthcare professionals (therapists, doctors, and nurses)	Survey the attitudes of healthcare professionals (doctors, nurses, therapists) and in-patient stroke patients after continuous IMU use	Method: Quantitative: Study-generated questionnaire * Response Type: Close-ended questionnaire with 7- and 10-point scale options * Qualitative: Interview (open-ended and close-ended questions) * ISO Usability: 3	Close-Ended Questions: Stroke: - Sensors were easy to operate and to learn to use, were comfortable, did not limit daily activities, and were unobtrusive in appearance Healthcare Professionals: - All agreed that sensors were easy to learn how to use and operate - Views differed regarding the opportunity for wearable tracking sensors and AI in healthcare delivery and whether the technology could be used without the control of a human caregiver - Only therapists viewed the increasing use of sensors and artificial intelligence technologies as an opportunity for healthcare applications Open-Ended Questions: Stroke: - Reported that sensors were easy to operate and learn to use, were comfortable, did not limit daily activities, did not cause anxiety, and were unobtrusive in appearance - Inpatients disliked the straps (caused irritation for some) and the size of the device - Expected the device to have heart rate and time functionalities Healthcare Professionals: - All viewed the system as not intrusive to healthcare - 10 highlighted sensors would only cause discomfort to a selection of patients in certain situations (e.g., some cases of hemiparesis, swelling or long wear periods) - Wanted the ability to combine data with other clinical measurements - Expected instructions on how to use the device
[37]	30 with stroke	Aim 1: Determine ease of understanding feedback from wearable sensor data (both arm/hand use and mobility) for chronic stroke survivors Aim 2: Identify stroke survivors’ preferences for feedback metrics (i.e., mode, content, frequency, and timing) that have the potential to drive health-supporting behavior change	Method: Quantitative: Study-generated questionnaire * Response Type: Likert scale Qualitative: Interviews * ISO Usability: 3	Quantitative: - Mobility metrics: Participants preferred hourly steps, followed by daily steps - Upper limb metrics: Participants preferred hourly arm use followed by daily arm use and arm use ratio Qualitative Themes: Motivation for behavior change - Feedback metrics were easier to understand once participants were oriented to them - Mobility metrics easier to understand than upper limb - Participants with severe motor impairments felt that they were using their paretic arm to max capacity and that additional feedback would not be helpful Need for real-time feedback based on individual goals - Participants preferred daily summary - Many preferred real-time feedback Value of guidance from experienced clinicians for prescription and accountability - Participants reported frequent meetings with therapists and clinicians would be more helpful for promoting behavior change, fostering accountability and offering strategies to facilitate movement during daily activities
[38]	30 with stroke	Establish short-term usability of wrist-worn wearable sensors to capture arm and hand movement behavior in the unsupervised home or community environment in people with chronic stroke	Method: Quantitative: System Usability Scale (SUS) * Qualitative: Interviews * ISO Usability: 1,2,3	Quantitative: - SUS 85.4 (SD = 13.0) Qualitative: - Reasons for lack of adherence were cold-like symptoms unrelated to the study (*n* = 2), forgetting to charge or wear the sensors (*n* = 3), or incompatible activities (*n* = 2) - Researchers walked participants through the procedures to resolve the synchronization issue; 2 participants did not understand this procedure - Challenges in donning and doffing the sensors were reported by those with severe motor impairments; 3 received regular assistance from caregivers - Participants reported knowledge of being monitored was a motivation to use their paretic arm and hand more

* Indicates questionnaire/interview guide is provided.

## Data Availability

No new data were created or analyzed in this study. Data sharing is not applicable to this article.

## Abbreviations

The following abbreviations are used in this manuscript:

6MWT	Six-Minute Walk Test
ADL	Activities of Daily Living
FAC	Functional Ambulatory Category
FMI	Fugl-Meyer Assessment
Hem	Hemorrhagic Stroke
IMU	Inertial Measurement Unit
Isch	Ischemic Stroke
ISNCSCI	International Standard for Neurological Classification of Spinal Cord Injury
ISO	International Organization of Standardization
MMSE	Mini Mental State Examination
MoCA	Montreal Cognitive Assessment
mRS	Modified Rankin Scale
QUEST	Quebec User Evaluation of Satisfaction with Assistive Technology
SUS	System Usability Scale
TIA	Transient Ischemic Attack

## Appendix A. Preferred Reporting Items for Systematic Reviews and Meta-Analyses Extension for Scoping Reviews (PRISMA-ScR) Checklist

**Section**	**Item**	**PRISMA-ScR Checklist Item**	**Reported on Page Number**
**Title**			
Title	1	Identify the report as a scoping review.	1
**Abstract**			
Structured summary	2	Provide a structured summary that includes (as applicable): background, objectives, eligibility criteria, sources of evidence, charting methods, results, and conclusions that relate to the review questions and objectives.	1
**Introduction**			
Rationale	3	Describe the rationale for the review in the context of what is already known. Explain why the review questions/objectives lend themselves to a scoping review approach.	1–2
Objectives	4	Provide an explicit statement of the questions and objectives being addressed with reference to their key elements (e.g., population or participants, concepts, and context) or other relevant key elements used to conceptualize the review questions and/or objectives.	2–3
**Methods**			
Protocol and registration	5	Indicate whether a review protocol exists; state if and where it can be accessed (e.g., a Web address); and if available, provide registration information, including the registration number.	3
Eligibility criteria	6	Specify characteristics of the sources of evidence used as eligibility criteria (e.g., years considered, language, and publication status), and provide a rationale.	3
Information sources *	7	Describe all information sources in the search (e.g., databases with dates of coverage and contact with authors to identify additional sources), as well as the date the most recent search was executed.	3
Search	8	Present the full electronic search strategy for at least 1 database, including any limits used, such that it could be repeated.	Appendix B
Selection of sources of evidence †	9	State the process for selecting sources of evidence (i.e., screening and eligibility) included in the scoping review.	3
Data charting process ‡	10	Describe the methods of charting data from the included sources of evidence (e.g., calibrated forms or forms that have been tested by the team before their use, and whether data charting was done independently or in duplicate) and any processes for obtaining and confirming data from investigators.	4
Data items	11	List and define all variables for which data were sought and any assumptions and simplifications made.	4
Critical appraisal of individual sources of evidence §	12	If done, provide a rationale for conducting a critical appraisal of included sources of evidence; describe the methods used and how this information was used in any data synthesis (if appropriate).	N/A
Synthesis of results	13	Describe the methods of handling and summarizing the data that were charted.	4
**Results**			
Selection of sources of evidence	14	Give numbers of sources of evidence screened, assessed for eligibility, and included in the review, with reasons for exclusions at each stage, ideally using a flow diagram.	4–5
Characteristics of sources of evidence	15	For each source of evidence, present characteristics for which data were charted and provide the citations.	7,8,11–19
Critical appraisal within sources of evidence	16	If done, present data on critical appraisal of included sources of evidence (see item 12).	N/A
Results of individual sources of evidence	17	For each included source of evidence, present the relevant data that were charted that relate to the review questions and objectives.	7,8,11–19
Synthesis of results	18	Summarize and/or present the charting results as they relate to the review questions and objectives.	5–6,9–10
**Discussion**			
Summary of evidence	19	Summarize the main results (including an overview of concepts, themes, and types of evidence available), link to the review questions and objectives, and consider the relevance to key groups.	20–22
Limitations	20	Discuss the limitations of the scoping review process.	22
Conclusions	21	Provide a general interpretation of the results with respect to the review questions and objectives, as well as potential implications and/or next steps.	22
**Funding**			
Funding	22	Describe sources of funding for the included sources of evidence, as well as sources of funding for the scoping review. Describe the role of the funders of the scoping review.	22
* Where *sources of evidence* (see second footnote) are compiled from, such as bibliographic databases, social media platforms, and Web sites. † A more inclusive/heterogeneous term used to account for the different types of evidence or data sources (e.g., quantitative and/or qualitative research, expert opinion, and policy documents) that may be eligible in a scoping review as opposed to only studies. This is not to be confused with *information sources* (see first footnote). ‡ The frameworks by Arksey and O’Malley (6) and Levac and colleagues (7) and the JBI guidance (4, 5) refer to the process of data extraction in a scoping review as data charting. § The process of systematically examining research evidence to assess its validity, results, and relevance before using it to inform a decision. This term is used for items 12 and 19 instead of “risk of bias” (which is more applicable to systematic reviews of interventions) to include and acknowledge the various sources of evidence that may be used in a scoping review (e.g., quantitative and/or qualitative research, expert opinion, and policy document).			

## Appendix B. Database Search Terms

The search was conducted using controlled vocabulary (e.g., MeSH terms) and keyword searches.

Embase
  1. Wearable Technology Terms
    1. wearable computer/
    2. (wearable electronic device* OR wearable sensor* OR inertial measurement unit*)
    3. 1 OR 2
  2. Stroke Terms
    4. exp cerebrovascular accident/
    5. (stroke* OR cerebrovascular accident* OR cerebral vascular accident* OR CVA*).
    6. 4 OR 5
  3. Final Search Combination
    7. 3 AND 6
Medline
  1. Stroke Terms
    1. exp Stroke/
    2. Stroke Rehabilitation/
    3. (stroke* OR cerebrovascular accident* OR cerebral vascular accident* OR CVA*)
    4. 1 OR 2 OR 3
  2. Wearable Technology Terms
    5. wearable electronic devices/OR fitness trackers/
    6. (wearable electronic device* OR inertial measurement unit* OR wearable sensor*)
    7. 5 OR 6
  3. Final Search Combination
    8. 4 AND 7
CINAHL
  1. Wearable Technology Terms
    S1. (MH “Wearable Sensors”) OR (MH “Accelerometers”)
    S2. Wearable device* or wearable sensor* or inertial measurement unit*
    S3. S1 or S2
  2. Stroke Terms
    S4. (MH “Stroke+”)
    S5. (MH “Stroke Patients”)
    S6. Stroke or cerebrovascular accident* or cva or cerebral vascular event*
    S7. S4 OR S5 or S6
  3. Final Search Combination
    S8. S3 and S7
